# A modified hot phenol-based protocol for high-purity Escherichia coli lipopolysaccharide extraction: Biochemical validation, stem cell cytotoxicity, and dose dependent multi-organ inflammation in a rat model

**DOI:** 10.1016/j.btre.2025.e00933

**Published:** 2025-10-17

**Authors:** Edris Vahdani, Ali Sepehrinezhad, Elham Hosseini, Saman Soleimanpour, Sajad Sahab Negah, Mohammad Ahanjan

**Affiliations:** a Department of Medical Microbiology, School of Medicine, Mazandaran University of Medical Sciences, Sari, Iran; b Neuroscience Research Center, Mashhad University of Medical Sciences, Mashhad, Iran; c Department of Neuroscience, Faculty of Medicine, Mashhad University of Medical Sciences, Mashhad, Iran; d Department of Microbiology and Virology, School of Medicine, Mashhad University of Medical Sciences, Iran; e Antimicrobial Resistance Research Center, Bu-Ali Research Institute, Mashhad University of Medical Sciences, Mashhad, Iran; f Multiple Sclerosis Research Center, Neuroscience Institute, Tehran University of Medical Sciences, Tehran, Iran; g Immunogenic Research Center, School of Medicine, Mazandaran University of Medical Sciences, Sari, Iran

**Keywords:** Lipopolysaccharide, *E. coli*, Modified hot phenol method, Inflammation, Organ damage, Embryonic neural stem cell, Rats

## Abstract

Gram-negative bacteria contain lipopolysaccharides (LPS) in their outer membrane, which induce strong inflammatory responses. Traditional LPS extraction methods often leave residual protein and nucleic acid contamination. These impurities interfere with downstream analyses and reduce reproducibility in biological studies. This study provides a modified hot phenol method combined with enzymatic treatments using proteinase K, DNase, RNase to extract pure LPS from *Escherichia coli*. Purity was confirmed by gel electrophoresis using Coomassie blue and silver nitrate staining. The biological activity of isolated LPS was tested on mesenchymal and embryonic neural stem cells, demonstrating decreased viability. In Wistar rats, LPS injection elevated serum IL-6 but not TNFα or IL-1β. Histological examinations indicated liver, kidney, brain, and colon tissue damage post-injection. Our results show that the modified hot phenol method efficiently produces high-purity, biologically active LPS suitable for both in vitro and in vivo inflammation studies, supporting research into inflammatory processes and associated diseases.

## Highlights

• A modified hot-phenol protocol with enzymatic treatment for high-purity *Escherichia coli* LPS extraction.
• First report linking this extraction method to cytotoxicity in both mesenchymal and embryonic neural stem cells.
• Validation of extracted LPS in a rat model shows dose-dependent multi-organ inflammation.
• Provides a fully validated, reliable LPS product for inflammation research from bench to bedside.

## 1 Introduction

Lipopolysaccharide (LPS), often known as endotoxins, is a structural component of the outer membrane of a variety of Gram-negative bacteria. In *E. coli* (*E. coli),* approximately 2 × 10^6^ LPS molecules cover nearly 75 % of the membrane surface and constitute 5–10 % of the dry bacterial weight [[1], [2], [3], [4]]. LPS consists of three distinct components: i) lipid A, which anchors the molecule in the outer membrane; ii) an oligosaccharide core linked to lipid A by 3-deoxy-d-manno‑oct-olsonic acid (Kdo); and iii) a polysaccharide chain known as the O-specific antigen that extends outward from the cell surface [5,6].

LPS interacts strongly with host immune receptors, particularly activates Toll-like receptor 4 (TLR4), leading to the activation of macrophages and lymphocytes and the subsequent production of pro-inflammatory cytokines such as TNFα, IL-1β, and IL-6 [[7], [8], [9], [10]]. In addition to its pro-inflammatory and immunostimulant properties, LPS plays a critical role in the pathogenesis of a wide range of disorders, including metabolic, cardiovascular, and liver diseases [11]. Furthermore, LPS can activate brain microglia and induce systemic inflammation [12]. This occurs as a result of an interaction between LPS and the TLR4/CD14 complex found on the surfaces of peripheral monocytes/macrophages and brain microglia. As a result, this contact activates nuclear factor-kappa B (NF-κB), which leads to the production of pro-inflammatory cytokines. The pathogenesis of Alzheimer's disease has been linked to this biological process [13]. The use of LPS in medical and biological research has attracted significant attention. This is due to well-documented effects of LPS on various physiological systems, specifically its capacity to induce inflammation in the brain and respiratory systems. LPS also plays a crucial role in disease advancement and immune system activation [9,11,[14], [15], [16], [17]].

As a result, researchers have expressed a significant interest in obtaining a refined and pure derivative of LPS, which can be accomplished by a number of extraction and purification approaches [18,19]. This study aimed to extract *E. coli* LPS using the hot phenol method (as proposed by Westphal [20]) and then purify it using various enzymes and modifications to optimize its biological functions. Furthermore, the study intends to investigate the effects of extracted LPS both in vivo and in vitro. Furthermore, the study intends to investigate the effects of extracted LPS both *in vivo* and in vitro.

## 2 Materials and methods

### 2.1 Ethical statements

The experimental procedures and approaches used in this study were conducted and documented in accordance with the recommendations provided by the Animal Research: Reporting of In Vivo Experiments (ARRIVE) guidelines [21] and received approval from the Animal Care and Use Committee (ACUC) at Mazandaran University of Medical Sciences, following the ethical code of IR.MAZUMS.REC.1402.15133, as per the National Institutes of Health guidelines [22].

### 2.2 Isolation and confirmation of *E. coli*

Clinical *E. coli* samples were obtained from the Department of Microbiology and Virology at Ghaem Hospital, Mashhad. Biochemical identification was performed using standard Enterobacteriaceae assays. Sugar fermentation was confirmed using Kligler Iron Agar (KIA) and Sulfide–Indole–Motility (SIM) media, while enzyme activities were evaluated through urease (Christensen’s medium) and citrate utilization (Simmons citrate medium) tests. Amino acid decarboxylation was assessed with Lysine Iron Agar (LIA) medium. The combined results confirmed the biochemical identity of *E. coli* isolates [23].

### 2.3 LPS extraction and purification

**Fig. 1:**
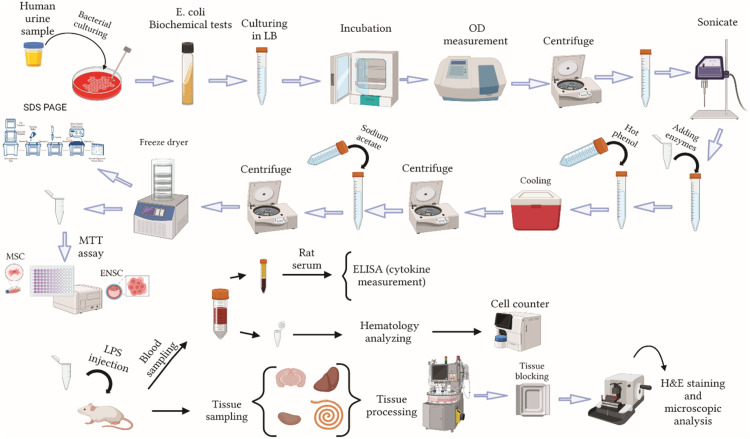
Schematic illustration of experimental protocol in different phases of the study. Created with BioRender.com.

Following the verification of the bacteria, one colony was taken out of the Mac-Conkey agar medium and dissolved in 5 ml of Luria broth (LB) medium, which was then added to a 15 ml Falcon [5]. After that, the falcons were put in a shaker incubator at a temperature and speed of 37 °C and 200 rpm, respectively. The bacterial suspension was taken out of the shaker incubator after 24 h and diluted 1:10 with LB. Then, 1.5 ml of the diluted suspension was placed in a spectrophotometer set to measure wavelengths of 600ʎ (OD600) and read the concentration. The bacterial suspension was centrifuged at 10,600 g for 10 min. After the supernatant was removed, the pellet was washed twice in phosphate-buffered saline (PBS) containing 0.15 mM CaCl_2_ and 0.5 mM MgCl_2_. The bacterial pellet was sonicated for 10 min in 10 ml of PBS [2,5]. Following treatment with proteinase K (150 μg.ml^−1^), the suspension was incubated for one hour at 65 °C. After adding RNase (45μg/ml), DNase (25 μg.ml^−1^), MgSo_4_ 20 % (1 μL.ml^−1^), and chloroform (4 μL.ml^−1^) to the suspension, it was incubated at 37 °C for 24 h. The next day, a similar volume of hot 90 % phenol was added to the mixture and it was vigorously agitated for 15 min at 65–70 °C. After cooling on ice, the suspension was moved to a 1.5 ml microtube. The mixture-filled microtubes were centrifuged for 15 min in 4 °C at 8500 g. After that, the supernatant was moved to a falcon 15 ml container. 300 microliters of sterile distilled water were added to the mixture in order to separate the phenol. To encourage LPS precipitation, the extract was further supplemented with 0.5 M sodium acetate and 95 % ethanol. It was then kept at −20 °C overnight (a precipitate with a cloud-like appearance should appear). Falcon was centrifuged in a refrigerator-operated centrifuge set at 4 °C and in 2000 g for 10 min. Following removing the supernatant, one milliliter of sterile distilled water was added to the precipitated pellet in order to completely remove the phenol. Precipitated LPS was then lyophilized and stored at 4 °C [[2], [3], [4], [5], [6], [7], [8], [9], [10], [11], [12], [13], [14], [15], [16], [17], [18], [19], [20], [21], [22], [23], [24], [25]] (Fig. 1).

### 2.4 Coomassie blue and silver staining

The purity of the extracted LPS was evaluated using polyacrylamide gel electrophoresis and staining with Coomassie blue and silver nitrates as methods previously described [26,27]. The LPS solution was mixed with 20 µL sample buffer and heated in a thermal cycler for 8 min at 95 °C (Protein denaturation). The sample mixture was then loaded to a 12 % separation and 4 % thickening polyacrylamide gel (10 µL per well) using a Hamilton syringe. To identify and determine the molecular weight of unknown samples, protein markers, also known as Low Molecular Weight (LMW) protein markers, have been developed. Following the loading of the sample onto the separation gel, the electrophoresis was first run at 120 V and then elevated to 170 V. After electrophoresis, Coomassie blue and silver nitrates staining proceeded to confirm the absence of protein contamination and visualization of the sample bands, respectively [28].

### 2.5 Embryonic neural stem cell culture

Embryonic neuronal stem cells (ENSCs) were obtained from brain biobank in the Department of Neuroscience at Mashhad University of Medical Sciences. Primary cultures ENSCs were prepared as previously described [29,30]. Following centrifugation, the cells were placed in 25cm^2^ culture dishes containing a customized medium containing the following ingredients: 1 % glutamine, 2.5 % fetal bovine serum (FBS), 20 ng.ml^−1^ epidermal growth factor (EGF), 1 % penicillin/streptomycin solution, 1 % N2 supplement, and B27 (Gibco). The cells were then incubated at 37 °C with 5 % CO2 in a humid atmosphere. The medium was changed every two or three days. When cells were approximately 80 % confluent, they were exposed to LPS before being investigated in greater detail.

### 2.6 Culture of mesenchymal stem cells

The rat's tibia and femur mesenchymal stem cells (MSCs) were obtained from brain biobank in the Department of Neuroscience at Mashhad University of Medical Sciences. The cells were gently thawed and transferred to a flask containing DMEM, 1 % glutamine, 1 % penicillin/streptomycin solution, and 10 % FBS (Germany, Gibco). The cells were then incubated at 37 °C with 5 % CO2 in a humid atmosphere. The medium was changed every two or three days [31].

### 2.7 Cell viability measurement (MTT test)

The effect of LPS on cell viability and proliferation was evaluated using the 3-[4,5-dimethylthiazol-2-yl] −2,5 diphenyl tetrazolium bromide (MTT) test (Sigma-Aldrich, Germany). About 1 × 10^4^ cells were seeded into each well of a 96-well plate, and the cells were left to stabilize for 24 h. After that, ENSCs and MSCs exposed to LPS at 800 μg.ml^−1^ and 700 ng.ml^−1^, respectively. Following a designated 72-hour test duration, 10 μL of MTT solution (1 mg.ml^−1^ in PBS) was introduced into each well, and the cells were then incubated for 4 h at 37 °C with 5 % CO2 and 95 % air. Subsequently, 100 μL of dimethyl sulfoxide (DMSO) was added to each well. The wells were shaken at 37 °C, and the absorbance at 570 nm was measured with a microplate spectrophotometer [32,33].

### 2.8 Experimental animal groups

Male Wistar rats weighing between 250 and 300 g were provided by the Animal Center of Mashhad University of Medical Sciences. The rats were kept in controlled conditions with access to food and water in compliance with ethical standards, and a researcher who had been blinded to prevent bias conducted some experiments. Based on previous studies, a total of 24 male Wistar rats were chosen for the present study. Four groups of these rats were randomly assigned; the control group and the other three groups had injections of different concentrations of LPS. There were six male rats in each group. Model groups received intraperitoneal dosages of LPS (5.5 mg.kg^−1^, 8 mg.kg^−1^, and 10 mg.kg^−1^) whereas the control-vehicle group received 250 µl of 0.9 % sodium chloride. After that, each rat was given its container and returned to the animal center. After 24 h, rats were anesthetized using a combination of 60 mg.kg^−1^ ketamine and 80 mg.kg^−1^ xylazine. Afterwards, target tissues and blood samples were collected.

### 2.9 Hematology tests

After the end of the experiment, rats were euthanized and blood samples were directly collected from the heart in tubes containing calcium ethylenediaminetetraacetic acid (EDTA; 10 % 0.5 M). The samples were quickly transferred to the laboratory and examined using a fully automatic hematology analyzer to measure various parameters, including hemoglobin level, hematocrit, number of red blood cells, number of leukocytes, number of platelets, mean corpuscular volume (MCV), average body hemoglobin (MCH), and mean corpuscular hemoglobin (MCHC) [34].

### 2.10 Preparing and staining of tissues for histopathological examinations

Samples of the colon, kidney, liver, and brain were taken from the various experimental groups, and were fixed for 72 h in solutions containing 10 % formalin in sodium chloride. The tissue was then subjected to additional processing and infusion with 70 % ethanol (Merck, Germany), to gradually increase its concentration to 100 % in order to remove any remaining water. After that, the tissue was cleaned and placed in xylene to enhance paraffin penetration. Subsequently, all tissues were incorporated into paraffin and the blocks were cut to a thickness of 6 µm using a microtome. All sections were dried at room temperature, deparaffinized, and hydrated by gradually reducing the concentration of ethanol in distilled water to prepare for subsequent staining. First, the sections were stained with hematoxylin and washed with tap water and distilled water. Finally, the samples were stained with eosin Y solution, washed with distilled water, and treated with increasing concentrations of ethanol before clearing with xylene [[34], [35], [36]].

### 2.11 Histopathological examinations

Liver injury was evaluated in nine fields of microscopic view in each section (magnification×40) and the mean scores which were previously identified were then taken into account to determine the liver injury scores for each group. All scores were examined as follows: 0, Intact liver without pathological findings; 1, hepatocyte vacuolation and focal nuclear pyknosis; 2, moderate injury, severe nuclear pyknosis, and cytoplasmic hypereosinophilia; 3, necrosis, hemorrhage, and neutrophil infiltration [34,37]. The kidney injury was scored based on previous studies as follows: 0, no damage to epithelial cells; Also, there are 4 grade of injury based on the extent of involved area with degenerative epithelial cells: 1, 25 % (mild damage); 2, 25 % to 50 % (moderate damage); 3, 50 % to 75 % (severe injury), 4, 75 % to 100 % (very severe damage) [38,39]. Ten light microscope fields were used to evaluate brain histological alterations. The percentage of vacuolated cells in the cerebral cortex of animals was examined as previously described [40]. Furthermore, the average number of inflammatory cells such as neutrophils and plasma cells were counted in 8 microscopic fields in lamina propria of intestinal tissues (magnification×100) [34].

### 2.12 Measurement of pro-inflammatory cytokines

After centrifuging the animals' blood at 2000 RPM for 10 min, the obtained serum was gently collected and stored at −70 °C for further analysis. The levels of pro-inflammatory cytokines (IL-1β, IL-6, and TNFα) in serum were measured using specialized ELISA kits based on the manufacturer's instructions (Karmania Pars Gene (KPG), Iran).

### 2.13 Statistical analysis

Data analysis in the present study was conducted using GraphPad Prism software. To identify significant differences between more than three experimental groups, a one-way analysis of variance (ANOVA) and the corresponding Tukey post-hoc test were performed. Unpaired *t*-test was conducted to compare cell viability between control and LPS groups. The results were expressed as mean ± standard deviation (SD), providing an accurate assessment of treatment effects and identification of any significant differences between experimental groups with confidence.

## 3 Results

### 3.1 Bacterial biochemical tests

**Fig. 2:**
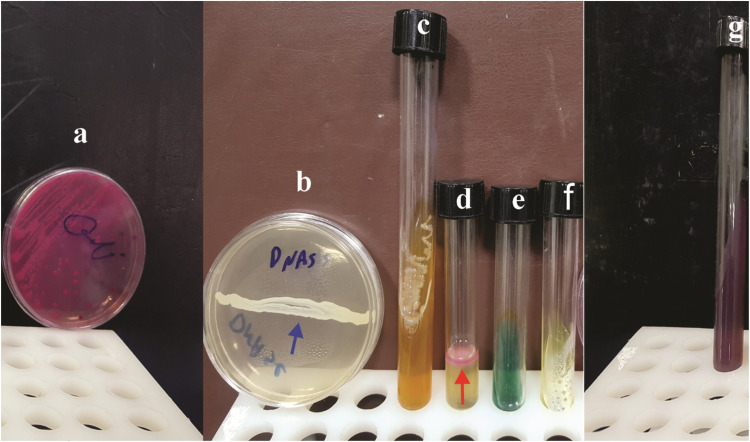
Enterobacteriaceae biochemical tests. (a) *E. coli* colonies on McConkey agar medium (flat, dry, pink colonies). (b) DNase agar: No halo was observed after adding of HCL to the bacterial culture line (blue arrow) (negative). (c) Glucose sugar fermentation and acid production at the surface and depth of the KIA medium, with no H_2_S formation (medium does not turn black). (d) SIM culture medium indicates the movement of bacteria throughout the length of the culture line and indole synthesis immediately after adding Coax reagent as a ring on the surface (red arrow). (e) Simon's citrate culture media has not changed color (green), indicating the lack of citratease enzyme. (f) The urea agar culture medium has not changed color and shows the lack of urease enzyme (negative). (g) The decarboxylase test is performed in a lysine decarboxylase broth medium to detect bacterial decarboxylation of lysine.

Several biochemical tests were conducted to identify the Enterobacteriaceae family and confirm the isolated *E. coli* strain. The results of culture on the KIA medium were acid/acid (A/A), the results of the H_2_S gas generation were negative, and the findings of motility and indole gas production following cultivation in the SIM medium were positive. Furthermore, the findings of the decarboxylation of amino acid lysine in Lysine decarboxylase test in lysine decarboxylase broth medium, as well as the results of the culture of the Simon citrate medium to evaluate the existence of the citrate enzyme and the usage of sodium citrate were negative. Moreover, the DNase medium cultivation and the Christian medium cultivation for urease enzyme monitoring yielded negative results (Fig. 2).

Following the biochemical confirmation of the *E. coli* strain, we proceeded with a sequential evaluation on the extracted LPS. The first and most important step was simply to confirm that it was biochemically pure. This means that it would not have any common contaminants, such as proteins, nucleic acids. Once high purity was established, we then investigated its functional biological activity. In this case, we first examined it in vitro for cytotoxicity on stem cells that are central mediators of inflammation and tissue repair. Finally, the purified LPS compound was injected into a rat model, to examine the pathological inflammatory response and multi-organ injury. Through this sequential process, we can reasonably with confidence attribute the biological effects to the LPS itself and not any residual contaminant.

### 3.2 SDS-PAGE results

**Fig. 3:**
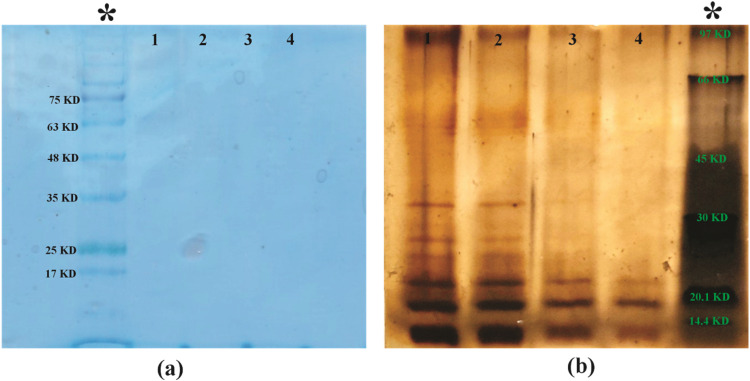
(a) Brilliant Coomassie Blue G250 and (b) silver nitrate polyacrylamide gel staining. Lines 1, 2, 3, and 4 indicate serial dilution of total extracted LPS that were isolated from *E. coli* (100 %, 75 %, 50 %, and 25 %, respectively). It is revealed that there are no bands in the Coomassie blue staining and that there are definite bands in the silver nitrate staining in the weight range of 20 to <45 KD. (*) stands for protein marker.

Polyacrylamide gel electrophoresis, using Brilliant Coomassie Blue G250 as a stain, is a technique for detecting proteins by forming visible bands in the gel (Fig. 3a). To ensure that the extracted LPS is devoid of protein impurities, the proteinase K enzyme is utilized during the extraction procedure. The silver nitrate staining method reveals three distinct bands ranging from 20 to 30 Kilo Dalton (KD), which correspond to the molecular weight of LPS. The bands in row 1 appear clearer than those in row 2, which could be due to the 100 % concentration of extracted LPS. Row 3, with a 50 % concentration, has two discernible bands at 20KD and slightly higher. Finally, row 4, with a 25 % concentration, shows only one clear band (Fig. 3b).

### 3.3 Cell viability

**Fig. 4:**
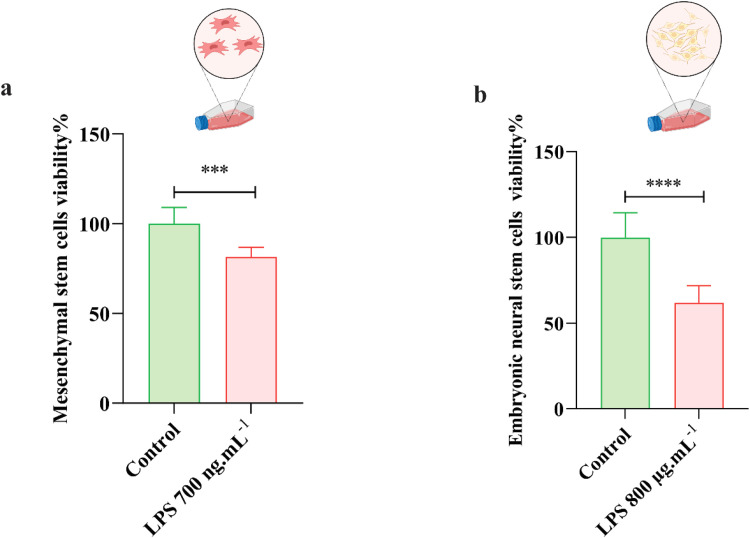
Effect of LPS on cell viability in two cell lines. The percentages of mesenchymal stem cells (*n* = 6) and embryonic neural stem cells (*n* = 6) that survive in the presence of LPS are shown in (a) and (b) respectively. All values are presented as mean ± SD.

The results of MTT assay indicated that the percentage of cell viability in mesenchymal stem cell cultures was statistically decreased 72 h after incubation with 700 ng.ml^−1^ of LPS extracted from *E. coli* (*P* < 0.001; Fig. 4a). Likewise, cell viability was significantly reduced in embryonic neural stem cell cultures after the same exposure with 800 μg.ml^−1^ of extracted LPS (*P* < 0.0001; Fig. 4b).

After established the cytotoxic potential of the extracted LPS in stem cell cultures, we wanted to know if these effects can be extrapolated to a more complex in vivo system. Thus, we evaluated the systemic inflammatory and pathological responses to LPS in a Wistar rat model.

### 3.4 Hematological changes

After anesthesia, blood samples were directly collected by cardiac puncture. Subsequently, an automated hematology analyzer was used to measure the hematological variables on these samples (Table 1). While all experimental rats showed a rise in the average number of circulating WBC, this parameter was only statistically significantly elevated in the 5.5 mg.kg^−1^ group compared to the control-vehicle group (*P* < 0.01). Surprisingly, the results also showed that the average number of blood platelets significantly decreased after injection of LPS in rats (*P* < 0.01). The difference in other hematological parameters was not statistically significant between LPS groups and sham (Table 1).

**Table 1:** Fully automated blood analysis of the experimental groups.

	Sham (control-vehicle)	LPS 5.5 mg.kg^−1^	LPS 8 mg.kg^−1^	LPS 10 mg.kg^−1^
WBC (×10^3^/mm^3^)	7.192 ± 1.38	12.61 ± 2.55*	10.56 ± 3.73	9.093 ± 3.44
RBC (×10^6^/mm^3^)	8.343 ± 0.23	8.897 ± 0.52	7.848 ± 1.12	8.583 ± 0.46
HGB (g/dl)	15.53 ± 0.52	17.05 ± 0.85	14.95 ± 2.24	15.90 ± 0.99
HCT ( %)	43.50 ± 1.18	47.68 ± 2.07	42.20 ± 6.20	44.82 ± 2.87
MCV (fl)	52.15 ± 1.26	53.68 ± 1.68	53.73 ± 1.42	52.22 ± 1.98
MCH (pg)	18.63 ± 0.50	19.18 ± 0.56	19.02 ± 0.54	18.53 ± 0.87
MCHC (g/dl)	35.70 ± 0.24	35.77 ± 1.24	35.42 ± 0.48	35.48 ± 0.50
PLT (×10^3^/mm^3^)	669.5 ± 102.9	251.6 ± 84.19****	168.2 ± 63.36****	157.0 ± 103.5****

Data expressed as Mean ± SD.

⁎ Compared to vehicle-treated control (*n* = 5–6). LPS: lipopolysaccharide; WBC: white blood cell; HGB: hemoglobin; MCV: mean corpuscular volume; MCH: mean corpuscular hemoglobin; MCHC: mean corpuscular hemoglobin concentration; PLT: Platelets.

### 3.5 Systemic inflammatory response

**Fig. 5:**
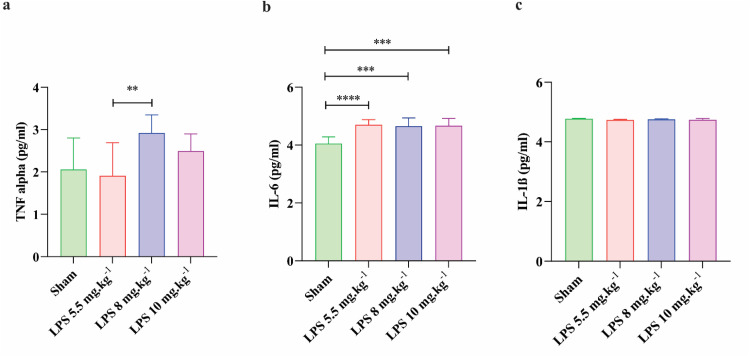
The effect of LPS on inflammatory responses in animals. The levels of pro-inflammatory cytokines TNFα, IL-6 and IL-1β in rat serum have been measured by ELISA kits (*n* = 6 rats per group). All values are presented as mean ± SD.

The study's findings showed that the average concentrations of TNFα were 2.05 ± 0.74, 1.91 ± 0.77, 2.92 ± 0.42, and 2.49 ± 0.40 pg.ml^−1^ in sham, LPS 5.5 mg.kg^−1^, LPS 8 mg.kg^−1^, and LPS 10 mg.kg^−1^, respectively. The levels of this pro-inflammatory cytokine were significantly higher at the 8mg/kg dose compared to 5.5 mg group (*P* < 0.01; Fig. 5a). TNFα concentrations did not differ significantly across other experimental groups. Also, the study found that the average concentration of IL-6 in sham, LPS 5.5 mg.kg^−1^, LPS 8 mg.kg^−1^, and LPS 10 mg.kg^−1^ were 4.05 ± 0.23, 4.70 ± 0.17, 4.65 ± 0.28 and 4.67 ± 0.24 pg.ml^−1^ respectively. The concentrations of this cytokine were dramatically increased in all LPS groups compared to sham (Fig. 5b). Furthermore, the findings indicated that the levels of IL-1β in circulation of sham, LPS 5.5 mg.kg^−1^, LPS 8 mg.kg^−1^, and LPS 10 mg.kg^−1^ were 4.77 ± 0.01, 4.73 ± 0.02, 4.75 ± 0.01, and 4.74 ± 0.03 pg.ml^−1^, respectively. The values of this cytokine were not statistically significant between experimental groups (Fig. 5c).

### 3.6 LPS induced multiorgan tissue damage in rats

**Fig. 6:**
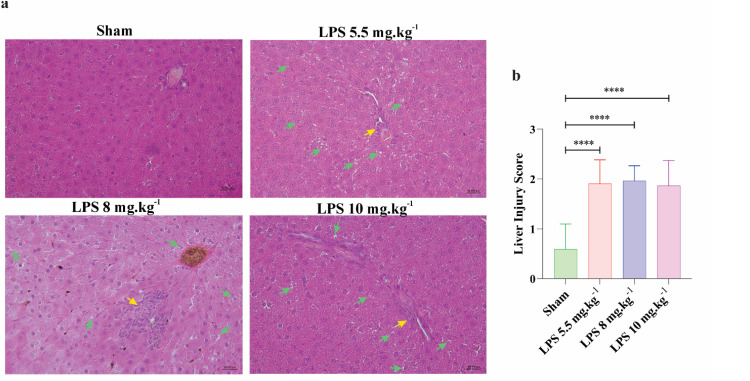
Histological changes of liver tissues in different experimental groups. (a) H&E staining of liver tissue (magnification×40) in experimental animals (*n* = 6 rats per group); The hepatocyte nuclear pyknosis is depicted by green arrows, while the infiltration of inflammatory cells into the liver tissue is indicated by yellow arrows. (b) The bar graph displays the average grade of liver injury in different experimental groups. All values are presented as mean ± SD.

The histological analysis of the liver tissues revealed hepatocyte nuclear pyknosis and increased the infiltration of circulating leukocytes in liver parenchyma in all LPS groups in comparison to control-vehicle rats (Fig. 6a). The average scores of liver injury were 0.59 ± 0.50, 1.90 ± 0.47, 1.96 ± 0.30, and 1.86 ± 0.50 in sham, LPS 5.5 mg.kg^−1^, LPS 8 mg.kg^−1^, and LPS 10 mg.kg^−1^, respectively. Overall, LPS, independent of dosage, increased total liver injury scores in experimental groups. (*P* < 0.0001; Fig. 6b).

**Fig. 7:**
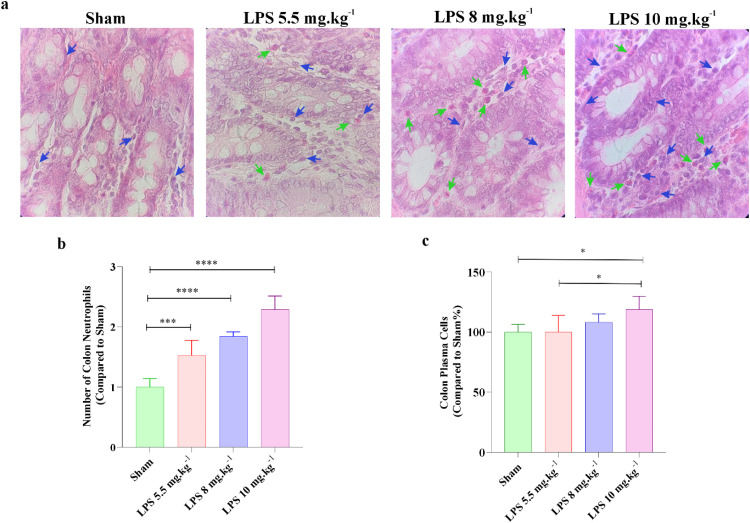
Histological changes of large intestine after injection of LPS in rats. (a) H&E staining of colon tissue (magnification×100) in different experimental animals (*n* = 6 rats per group); green arrows indicate infiltrated neutrophils and blue arrows display plasma cells in the lamina propria. (b) The bar graphs display the average number of neutrophils and (c) plasma cells in the large intestine. All values are presented as mean ± SD.

The histological analysis of the colon showed that injection of LPS statistically increased the number of neutrophils in lamina propria of large intestines in all experimental rats (Fig. 7a and b). However, the number of plasma cells was only increased in the lamina propria of 10 mg.kg^−1^ rats as compared to the sham group (*P* < 0.05; Fig. 7a and c).

**Fig. 8:**
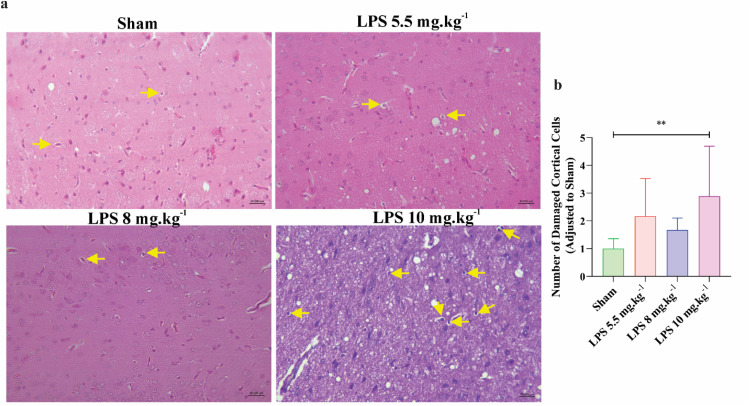
Histological alterations of cerebral cortex in experimental groups. (a) H&E staining of brain tissue (magnification×40) in different experimental animals (*n* = 6 rats per group). The yellow arrows indicate vacuolated and swollen cells. (b) The bar graph displays the average number of swollen cells. All values are presented as mean ± SD.

Histological examinations of cerebral tissues showed that the number of swollen cells was significantly increased in the cerebral cortex of 10 mg.kg^−1^ rats as compared to the control-vehicle group (*P* < 0.01; Fig. 8a and b). However, the alterations of the cerebral cortex were not statistically significant in other LPS groups when compared to control rats (Fig. 8).

**Fig. 9:**
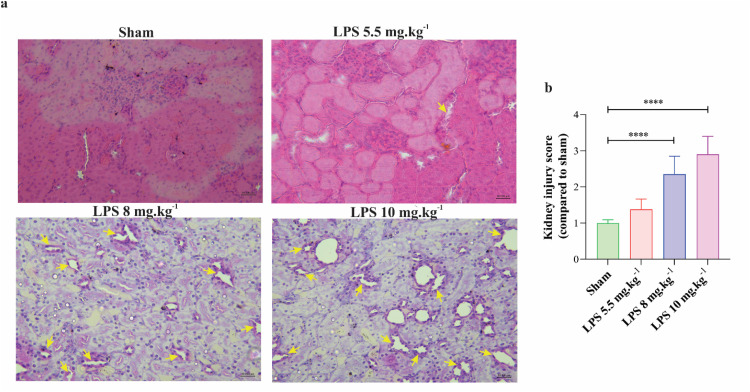
Histological observations of kidney tissues in rats after injection of LPS. (a) H&E staining of Kidney tissue (magnification×40) in different experimental animals (*n* = 6 rats per group); the yellow arrows indicate degenerated tubular epithelial cells. (b) The bar graph displays the average grade of kidney injury in different experimental groups. All values are presented as mean ± SD.

Histological evaluations of kidney tissues demonstrated an increase in the regions containing degenerative tubular epithelial cells at dosages of 8 mg.kg^−1^ and 10 mg.kg^−1^ when compared to sham, but no statistically significant alterations were seen in the 5.5 mg.kg^−1^ group (Fig. 9).

## 4 Discussion

The majority of gram-negative bacteria's plasma membranes require LPS to provide structural integrity and this structural molecule is what causes septic shocks and inflammatory disorders after bacterial infections. Different immune cells produce pro-inflammatory cytokines when triggered by LPS [2]. The inflammatory effects of LPS are primarily caused by the activation of the TLR4 signaling pathway in the central nervous system [41]. LPS has been extracted using a variety of procedures to ensure its purity and biological activity. Trichloroacetic acid, butanol without water, triton/Mg^+2^, ether without water, methanol/petroleum-free ether chromosome, EDTA method, and hot phenol extraction method are all included in the previously described processes.

Even with potentially toxic elements like phenol, the most efficient technology is still the phenol thermal extraction method [42,43]. One of the major disadvantages in phenol-chloroform-petroleum ether (PCP) method is the loss of large levels of smooth LPS from the primary extracted source. The hot phenol technique does not exhibit this issue and is considered a reliable method for extracting pure LPS. However, the fundamental limitation of the hot phenol extraction method is contamination with other undesired substances such as proteins, capsular polysaccharides, and nucleic acids [44,45]. In the current study, we used hot phenol method with certain enzymatic modifications to overcome this basic limitation in extracting LPS from *E. coli*. In our modified method, following the sonication process, we treated the bacterial suspension with high concentrations of proteinase K (150 μg.ml^−1^), RNase (45μg/ml^−1^), and DNase (25 μg.ml^−1^) to ensure that any undesirable contaminants were removed. In the next step, remaining enzymes were eliminated from the solution using hot phenol. Pure LPS was then extracted after two rounds of phenol washing with sterile distilled water. Finally, extracted LPS was validated by SDS electrophoresis followed by Coomassie blue and silver staining.

Furthermore, the biological functions of extracted LPS were evaluated in vitro in ENSCs and MSCs as well as in vivo in Wistar rats. MSCs are multipotent stem cells that have therapeutic potential due to their ability to self-renew and differentiate into a variety of cell types [46]. ENSCs are also multipotent stem cells with the ability to self-renew and proliferate into a variety of neurons and glial cells [47]. In cell therapy, exposing these stem cells in inflammatory environments, such as LPS-rich medium, provides as a model for investigating their secretory and behavioral responses in inflammatory diseases [48,49]. The results of our study showed that the extracted LPS considerably decreased cell viability in both ENSC and MSC cultures after 72 h. These results are consistent with studies that demonstrated that LPS dramatically reduced cell viability and promoted apoptosis in MSC cells by activating caspase as well as increased cell cytotoxicity in NSCs [49,50]. The administration of LPS is a valid and common model for studying the pathophysiology and cellular mechanisms involved in inflammatory diseases [[51], [52], [53], [54], [55]].

The variation in LPS sensitivity observed between MSCs and ENSCs, in response to different dosages of LPS, may be explained by differences in the TLR4 complex expression in these cells. Rat bone-marrow-derived MSCs consistently express TLR4 and induce robust activation of NF-κB and MAPK signaling cascades in response to LPS, resulting in the secretion of TNFα, IL-6, and IL-8 and modulation of differentiation capacity [[56], [57], [58]]. ENSCs are also TLR4 positive but generally express lower basal levels of this receptor. Following LPS stimulation in this cell type, an overall reduction in proliferation and neuronal differentiation primarily answers to TLR4 stimulation, rather than producing a strong cytokine response [[59], [60], [61]].

We injected three different doses of extracted LPS in Wistar rats to evaluate systemic inflammatory responses and histopathological changes of multiple organs including the large intestine, liver, kidney, and cerebral cortex. The findings of the study showed that a distinct systemic inflammatory profile at 24 h following LPS injection, characterized by a significant increase of IL-6 but not in TNFα or IL-1β in all treatment groups. This interesting group of cytokines provides valuable insight into the kinetics of LPS-induced inflammation and the specific activity of our extracted LPS. The observed pattern aligns well with the established hierarchical and time course of cytokine release [[62], [63], [64], [65], [66], [67]]. TNFα and IL-1β are generally classified as early-response cytokines, secreted quickly by macrophages and monocytes, and their serum levels increase significantly within 1–4 h after LPS challenge, then they return to baseline [[68], [69], [70], [71], [72]]. In contrast, IL-6 is a major secondary cytokine and its production is often stimulated by TNFα and IL-1β, producing a more pronounced and sustained peak at around 24 h [73]. Therefore, our findings strongly suggest that the 24-hour time point captured the resolution phase of the early cytokine storm (TNFα/IL-1β) while highlighting the peak or sustained plateau of IL-6-driven inflammation. The IL-6 driven response is especially important, given its role in mediating an acute phase response, fever, and lymphocyte activation, all features of a prolonged inflammatory response [74]. The specific dose, timing of measurement, and the purity of our LPS preparation likely influenced this specific cytokine profile, highlighting the complexity of the immune response induced by a defined inflammatory trigger.

Surprisingly, our extracted LPS by modified hot phenol technique displayed severe thrombocytopenia 24 h after injection in rats. One mechanism that explains why LPS reduces the number of circulating platelets is the trapping of these cellular fragments in various organs such as the liver, lung, and spleen, which is mediated by tissue macrophages [75,76]. The second mechanism is associated with platelet activation via the TLR4-MyD88-cGMP-protein kinase G (PKG) pathway, which exacerbated platelet aggregation and caused thrombocytopenia in response to injection of LPS [77].

One interesting result from our study was that circulating WBCs significantly increased in the low-dose LPS group (5.5 mg.kg⁻¹), while we did not observe this response in the higher doses (8 and 10 mg.kg⁻¹). This non-linear relationship may be the result of the dynamic pathophysiology of severe systemic inflammation. The initial leukocytosis observed at the lower dose is a typical immune response, which is due to the bone marrow releasing leukocytes into circulation [78,79]. Nevertheless, at higher and more severe doses of LPS, a different phenomenon dominates, as global endothelial activation leads to upregulation of adhesion molecules such as selectins and integrins [80,81]. This promotes leukocyte apoptosis and the robust margination and sequestration of activated leukocytes away from circulation into peripheral tissues [[82], [83], [84], [85], [86], [87]], a process critical for the extensive organ infiltration we observed histologically in the liver and colon. This suggests that at 24 h post-injection, the robust extravasation of leukocytes into damaged tissues in the high-dose groups may have offset their release from the bone marrow, leading to the observed normalization of circulating counts. This hypothesis of dose-dependent leukocyte trafficking provides a coherent integration of our hematological and histopathological data.

We also showed that injecting the extracted LPS significantly induced multiple organ damages in animals, including hepatocyte nuclear pyknosis and infiltration of inflammatory cells into the liver parenchyma, increased inflammatory cells in lamina propria of the large intestine, promotion of swollen cells in cerebral cortex, and renal tubular epithelial cells degeneration. These findings are consistent with previous studies, as follows. Intraperitoneally injection of LPS induced liver inflammatory score in mice and rats [[88], [89], [90], [91]]. LPS activates TLR4 on Kupffer cells through lipopolysaccharide binding protein and CD14 surface receptors. These activated cells produce pro-inflammatory cytokines, which in turn induce liver injury [[92], [93], [94]]. Administration of LPS significantly induced inflammation, promoted apoptosis and changed the structural shape of the intestines in mice and rats [89,[95], [96], [97], [98], [99]]. LPS increased the permeability of intestinal barrier by activating and colonizing TLR4 and CD14 surface receptors on enterocytes and NF-κB signaling pathway [98,100]. Moreover, LPS induces inflammatory conditions by activating TLR4 on innate immune cells in the intestine [101]. In rodents, injection of LPS induces neuroinflammation, cellular swelling, microglial activation and, cellular necrosis in brain tissues [[40], [41], [42], [43], [44], [45], [46], [47], [48], [49], [50], [51], [52], [53], [54], [55], [56], [57], [58], [59], [60], [61], [62], [63], [64], [65], [66], [67], [68], [69], [70], [71], [72], [73], [74], [75], [76], [77], [78], [79], [80], [81], [82], [83], [84], [85], [86], [87], [88], [89], [90], [91], [92], [93], [94], [95], [96], [97], [98], [99], [100], [101], [102], [103]]. LPS can directly affect tight junction proteins and disrupt the integrity of the blood-brain barrier through several mechanisms such as activation of NF-κB, production of cyclooxygenase-2, activation of p38 mitogen-activated protein kinase, and overexpressing matrix metalloproteinases [104]. In the cerebral tissue, LPS interacts with TLR4 on microglia leading to the production of pro-inflammatory cytokines, secretion of matrix metalloproteinase 9 and generation of reactive oxygen species (ROS) results in neuroinflammation [105,106]. In mice, LPS significantly increase the vacuolated tubular epithelial cells in kidney [39,107]. LPS causes kidney injury by activating TLR4, which then expresses downstream signaling molecules including NF-κB and ROS, leading to the production of pro-inflammatory cytokines and apoptosis in renal tubules [39,108].

A key strength and originality of this work lies in its comprehensive validation strategy. While most studies that extract LPS focus on the biochemical purity, in this study we brought that important first step into strong functional validation using both in vitro and in vivo models. We show that LPS extracted from *E. coli* is not only pure, but biologically active, leading to significant cytotoxicity in stem cells and a dose-dependent multi-organ inflammatory response in a rat model. This dual approach connects a refined molecular method to biological outcomes and allows for a more complete and physiologically relevant assessment of the functionality of the extracted product.

The current study had some limitations. When measuring LPS purity, we relied on SDS-PAGE analysis with Coomassie blue and silver staining to show the absence of protein and the existence of characteristic LPS bands. While this was sufficient, additional and quantitative methods in future studies would strengthen the findings. For example, measuring endotoxin units using a Limulus Amebocyte Lysate (LAL) assay would provide a more rigorous and standardized method of assessing biological activity and purity. As noted by other methodological studies, using a multi-modal validation approach is crucial for comprehensively characterization of complex biological extracts and ensuring the reliability of downstream applications [25,109]. Analyzing the pro-inflammatory profiles of triggered cell lines and comparing the purity of extracted LPS with a standard sample using high-performance liquid chromatography (HPLC) may also provide further information about the efficacy of produced extracted. Furthermore, while enzymatic treatment with DNase and RNase was used to remove nucleic acids, but no quantitative validation including spectrophotometric A260/A280 measurement was used to confirm their removal represents another limitation of the study.

## 5 Conclusions

Our study demonstrates that LPS extracted from *E. coli* using the modified hot phenol method has a molecular weight range of 18 kDa to <45 kDa. The extracted LPS significantly inhibited cell proliferation in both MSCs and ENSCs, highlighting its functional impact. Additionally, it exacerbated systemic inflammation and caused multiorgan damage, with pathological effects observed in the gut, liver, kidney, and brain tissues. This extraction method yields LPS with strong inflammatory activity, establishing it as a reliable tool for in vitro and in vivo studies on inflammation and its associated disorders.

## Funding

This study has been supported by the Mazandaran university of Medical Sciences, Sari, Iran (ethical code: IR.MAZUMS.REC.1402.15133) for the fulfilment of Master of Science degree in Medical Bacteriology by E.V.

## Ethical approval

All the experimental procedures were performed in accordance with the ethical standards as recommended by the Animal Research: Reporting of In Vivo Experiments (ARRIVE) guidelines and received approval from the Animal Care and Use Committee (ACUC) at Mazandaran University of Medical Sciences, following the ethical code of IR.MAZUMS.REC.1402.15133.

## CRediT authorship contribution statement

**Edris Vahdani:** Writing – original draft, Visualization, Methodology, Investigation, Formal analysis, Data curation, Conceptualization. **Ali Sepehrinezhad:** Writing – original draft, Visualization, Methodology, Data curation, Conceptualization. **Elham Hosseini:** Writing – original draft, Data curation. **Saman Soleimanpour:** Writing – review & editing, Writing – original draft, Data curation, Conceptualization. **Sajad Sahab Negah:** Writing – review & editing, Writing – original draft, Data curation, Conceptualization. **Mohammad Ahanjan:** Writing – review & editing, Writing – original draft, Supervision, Methodology, Investigation, Funding acquisition, Data curation, Conceptualization.

## Declaration of competing interest

The authors declare that they have no known competing financial interests or personal relationships that could have appeared to influence the work reported in this paper.

## Data Availability

The datasets generated during and/or analyzed during the current study are available from the corresponding author on reasonable request.
